# Development of Quantitative Real-Time PCR Tests for the Identification of Biting Midge Species and Clades (Diptera: Ceratopogonidae) of the Obsoletus Group (Subgenus *Avaritia*), Including Important Viral Vectors in Europe

**DOI:** 10.3390/insects16050500

**Published:** 2025-05-07

**Authors:** Oliver Dähn, Bernd Hoffmann, Doreen Werner, Bruno Mathieu, Helge Kampen

**Affiliations:** 1 Friedrich-Loeffler-Institut (FLI), Federal Research Institute for Animal Health, Südufer 10, 17493 Greifswald, Germany; 2 Leibniz Centre for Agricultural Landscape Research (ZALF), Eberswalder Str. 84, 15374 Müncheberg, Germany; 3 Institutes of Bacteriology and Parasitology, Medical Faculty, University of Strasbourg, UR 3073 PHAVI, 67000 Strasbourg, France

**Keywords:** *Culicoides*, Obsoletus Group, *Avaritia*, vectors, real-time PCR, BTV, SBV, EHDV, COI, clades

## Abstract

Species of the widespread Obsoletus Complex (*Culicoides* subgenus *Avarita* Fox, 1955) have been implicated as potential key vectors during the bluetongue and Schmallenberg epidemics in Central Europe in 2006 and 2012. Although extensive efforts have been made to clarify vector–pathogen relationships, one of the most important steps in this process—correct species identification—remains difficult, due to the presence of isomorphic species within the Obsoletus Group. To overcome the difficulties in morphological species identification, several PCR tests were developed. With the aim of developing a high-throughput PCR, capable of differentiating all putative vector species and newly described haplotypes of the subgenus *Avaritia* present in Europe, a dataset of 4407 published sequences of the mitochondrial (mt) cytochrome c oxidase subunit I (COI) was used to develop specific primers and probes, which can either be applied in a singleplex PCR or in different multiplex PCR approaches. The real-time PCR achieved very high diagnostic sensitivity (100%) and specificity (91.7%) and reliably detected the three clades of *C. obsoletus* sensu stricto (s.s.) in a pool of specimens. Thus, the new real-time PCR approach will provide an excellent tool for large-scale monitoring, which could improve the understanding of the biology, geographical distribution, and habitat preference of European biting midge species involved in the transmission of bluetongue, Schmallenberg, and epizootic hemorrhagic disease viruses.

## Simple Summary

Biting midges of the genus *Culicoides* are small hematophagous flies, responsible for the spread of economically important arboviruses such as bluetongue virus. In Europe, the species of the Obsoletus Group have been identified as the main vectors, but the knowledge of the geographical distribution and ecology of many taxa of this group is scarce, not least of all because of missing identification tools. To address this issue, a high-throughput PCR approach was developed that allows the simultaneous examination of large quantities of individuals for the presence of isomorphic Obsoletus Group taxa, even in a pool of unsorted insects. In this work, the difficulties in the development of the PCR are addressed. The unresolved phylogeny of certain taxa and the difficulty in obtaining suitable DNA material for PCR validation have posed significant obstacles. Despite these challenges, the new PCR approach proved its potential by accurately identifying their target taxa. This establishes a crucial foundation for the routine implementation of surveillance studies, enabling detailed insights into the spatiotemporal distribution of vector species. As a result, current and future risk scenarios can be assessed, and countermeasures can be initiated to prevent viral spread. Especially in times of increasing globalization and a rapidly changing climate, this helps protect livestock and mitigate economic losses.

## 1. Introduction

Hematophagous insects are important vectors of disease agents worldwide, particularly species belonging to the dipteran families Culicidae, Ceratopogonidae, Psychodidae, and Simuliidae [1,2]. Contrary to mosquitoes (Diptera: Culicidae), which are certainly the most prominent representatives of this group of arthropods, biting midges of the genus *Culicoides* Latreille, 1809 (Diptera: Ceratopogonidae) have received much less public attention, even though they have been identified as vectors of filarial nematodes, protozoan parasites, and more than 50 viruses of veterinary and medical importance [3,4].

In Europe, indigenous biting midge species had been neglected as potent vectors until the unexpected emergence and fulminant spread of bluetongue virus (BTV) in several countries outside of the known distribution area of its main vector *C. imicola*. BTV can cause severe infection in ruminants, especially sheep [5,6]. The bluetongue disease (BTD) has a long history in Africa [7] and was initially reported in South Africa in the late eighteenth century [8]. Meanwhile, BTD has become endemic in the Mediterranean basin [9,10], and occasional spreading events take place in Central Europe, as recently observed during the incursion of BTV serotype 3 (BTV-3) and BTV-12 [11,12,13,14,15]. With the emergence of these virus strains, around one-third of all yet-known serotypes (13 of 36) [16] have already been confirmed for Europe [17] but the accessibility of effective vaccines [18] has so far prevented the disease from becoming endemic. The closely related epizootic hemorrhagic disease virus (EHDV) of the genus *Orbivirus*, on the other hand, was first described in North America where it was isolated from a wild-tailed deer [19]. It had never been reported in Europe until the recent isolation of EHDV-8 from infected farm animals in Italy (Sicily, Sardinia) and southern Spain (Andalusia) at the end of 2022 [20]. A high genetic similarity between the virus strain from Italy to the EHDV strains circulating in Tunisia in 2021 and 2022 suggests its introduction via the North African route [20], as also suspected for BTV in 2006 [21]. In the meantime, EHDV-8 has also been detected in Portugal and southern France [22]. In contrast to BTV and EHDV, nothing is known about the origin of the ruminant-pathogenic *Orthobunyavirus* Schmallenberg virus (SBV). Since its first appearance near the German–Dutch border in 2011, it has never completely disappeared [23] and rapidly established an enzootic status in many European countries [24,25].

Several native biting midge species have been implicated as potential vectors of these viruses, although true evidence of vector competence is scarce due to difficulties in rearing them under laboratory conditions. However, initial infection studies investigating the susceptibility of field-caught *Culicoides* to BTV [26,27] and EHDV [28,29] and virus isolations from field-collected specimens identified members of the Obsoletus Group (subgenus *Avaritia* Fox, 1955) as the main vectors of BTV [30,31,32,33,34,35], SBV [36,37,38,39,40,41,42,43,44,45,46,47], and EHDV [48].

In Europe, the Obsoletus Group was believed to consist of five species, including the eponymous *C. obsoletus* Meigen, *C. scoticus* Downes & Kettle, *C. chiopterus* (Meigen, 1830), *C. dewulfi* Goetghebuer, 1936, and *C. montanus* Shakirzjanova [49,50]. However, there are still taxonomic obscurities that have not yet been resolved completely, highlighting the ongoing challenge of identifying endemic vector species. Due to the analysis of various genetic markers, *C. dewulfi* has been excluded from the Obsoletus Group [51,52,53,54,55], while the phylogenetic status of *C. chiopterus* is still ambiguous. Depending on the gene markers analyzed, the latter is assigned to the Obsoletus Group in some cases (COI, ITS-1) [56,57] and excluded in others (28S rDNA) [56]. The remaining three taxa, *C. obsoletus*, *C. scoticus*, and *C. montanus*, are closely related species whose isomorphic females cannot be distinguished based on their morphological features. To facilitate morphological classification, these species are collectively referred to as ‘Obsoletus Complex’ or ‘Obsoletus/Scoticus Complex’ [58]. *Culicoides montanus* was thought to only be present further south in Europe [49]. A recent study reviewed all available COI sequences of subgenus *Avaritia* species published in GenBank, and the re-classification of some sequences revealed a much more northern distribution (UK, Germany, Poland) of the species [59]. Its taxonomic status within the Obsoletus Complex is still controversially discussed. Some authors propose that *C. montanus* might be a genetic variant of *C. obsoletus* rather than a separate species due to very low differences in the DNA sequence at certain gene loci [58,60]. Additionally, over the past two decades, further genetic variants of *C. obsoletus* [51,61,62,63] and *C. scoticus* [54,58] have been identified*,* with increasing evidence for *C. obsoletus* clades O1, O2, and O3 representing independent species [59].

All these native vector species feed on livestock and find excellent conditions for the development of their larvae in the surroundings of livestock farms [64], which significantly increases the risk of spreading BTV, SBV, and EHDV. *Culicoides obsoletus* and *C. scoticus* are generalists with opportunistic feeding behavior whose larvae can be found in a wide range of habitats such as forest litter, tree holes, silage residue, or manure [65]. *Culicoides chiopterus* and *C. dewulfi* almost exclusively take their blood meal from cattle and are strongly adapted to cattle dung as a breeding substrate [64,66,67]. Given this, along with the high density of animal farms and the extensive livestock trade within the European Union, it is not surprising that culicoid-borne pathogens spread and establish quickly, with considerable consequences for livestock industries. Although the dimensions of direct and indirect economic losses are difficult to assess [64], the financial impact e.g., of BTV-8 in Germany alone is estimated to be between 157 and 203 million Euro from the first cases in 2006 to the re-emergence in 2018 [68]. These immense costs emphasize the importance of effective vector management, but the required knowledge of the biology, ecology, and spatiotemporal distribution of native biting midge species is still limited.

One of the main reasons for these knowledge gaps is the costly and time-intensive nature of conducting surveillance studies, which rely on the consistent cooperation of selected livestock farms, skilled scientific personnel, and entomologists with years of experience to accurately identify captured biting midges. The identification of adult *Culicoides* is usually performed based on their morphology [54,69] but is rarely achievable for the isomorphic specimens of the Obsoletus Group [58,70].

Accordingly, for the reliable identification of these taxa, the development of different molecular methods has been the subject of numerous studies (e.g., [71,72,73]). By far the most widely used molecular techniques are those based on DNA amplification through polymerase chain reaction (PCR) [69]. Meanwhile, direct sequence analysis (DNA barcoding) of several mitochondrial (e.g., COI, 18S rRNA, cytb), ribosomal (ITS1, ITS2), and nuclear (e.g., CAD) markers has become a standard procedure for species identification and phylogenetic studies within the genus *Culicoides* [69]. However, a major disadvantage of DNA barcoding is the need to compare generated sequences with public genome databases such as GenBank, which have been shown to contain a substantial number of incorrect entries [59,74]. Additionally, the method allows only a single specimen to be processed per reaction tube [74], posing a major limitation for surveillance studies that often collect thousands of individuals per night [69]. This may be particularly problematic if species of the Obsoletus Group constitute a significant proportion of the captured specimens [73,75]. Hence, several medium-throughput multiplex PCR assays have been developed for the identification of the different Obsoletus Group members (e.g., [57,70,76,77]). However, most of these PCR tests are relatively complex to perform and analyze, and they are generally limited to specialized research groups, as they require the pre-sorting of captured insects and depend on time-consuming gel electrophoresis. To address at least some of these challenges, conventional multiplex PCRs for identifying almost all west Palearctic taxa of the subgenera *Culicoides* and *Avaritia* were recently developed [59,74]. The PCR tests are easy to analyze (producing one specific band per taxon) and can be run simultaneously using a standardized PCR protocol. While these PCR tests enable the analysis of multiple individuals in a pooled sample, they still require the morphological pre-sorting of catches at least to the group or complex level, which is why these tests are rather unsuitable for high-throughput screening.

A solution to this bottleneck may be provided using real-time PCR tests that are capable of identifying pooled *Culicoides* specimens, but only three of these assays have been developed so far for the differentiation of specimens belonging to the Obsoletus Group/Complex [62,73,78]. Of these assays, one was developed for the determination of the relative proportion of species in a pool of insects [73], which would make the analysis of large *Culicoides* collections to the species level viable [69]. However, this PCR is not up to date as it does not consider the different clades of *C. obsoletus*, whose geographical distribution and role in the spread of BTV, SBV, and EHDV is unknown. To address this issue, a real-time PCR approach was developed in this study that considers the latest taxonomic findings and is intended to quantitatively detect all potential vector species in unsorted pool samples.

## 2. Materials and Methods

### 2.1. Insect Collection and Morphologic Pre-Identification

Biting midges and non-culicoid Diptera (bycatch) were trapped year-round, both inside and outside livestock holdings, using BG-sentinel UV-light suction traps (Biogents, Regensburg, Germany), primarily in the framework of a Germany-wide monitoring project conducted from 2019 to 2022. Individuals of a few subgenus *Avarita* taxa have not been found in the collections and were obtained from other samplings in different European countries, Russia, and the USA. All captured midges were morphologically pre-identified at least to the group or complex level using common identification keys [79,80,81,82] and kept in 75% EtOH for further molecular analysis.

### 2.2. Extraction of Genomic DNA

DNA extraction was performed using either whole insects or parts of them (thorax, abdomen) (Table S1). For DNA extraction from whole specimens, the insects were removed from the storage vessel and placed on a clean paper tissue. After evaporation of the remaining preservative for 1 min at room temperature, the specimens were individually transferred to a clean 2 mL reaction tube containing either 180 µL buffer ATL and 20 µL proteinase K (QIAamp DNA Mini Kit) or 350 µL of house-intern ZB5d medium supplemented with antibiotics as described in Ries et al. [83]. Three steel beads with a diameter of 3 mm (TIS GmbH, Gauting, Germany) were added, and samples were completely homogenized for 3 min at 30 Hz using a TissueLyser II device (Qiagen, Hilden, Germany). Total genomic DNA was isolated with the QIAamp DNA Mini Kit (Qiagen) from ATL/Proteinase K containing homogenate in a 50 µL elution volume or with the NucleoMag VET Kit (Macherey-Nagel, Düren, Germany) from samples agitated in ZB5d/antibiotics mixture and finally eluted in 100 µL VEL-buffer. For DNA extraction from insect abdomens, total abdomens were removed from the insects and directly transferred to 200 µL buffer ATL/proteinase K mixture for further processing, as mentioned above. In some cases, DNA available from previous extractions (thorax and partial abdomen) [53,55] was directly used.

### 2.3. DNA Amplification and Sequencing

DNA extracts of single specimens or their body parts were used for the amplification of taxon-specific COI gene fragments, as described [59]. In total, 125 extracts were used for PCR amplification. Generated PCR products were subjected to agarose gel electrophoresis, and ethidium bromide-stained DNA bands of expected length (517 bp or 685 bp) were excised from the gels. The amplicons were recovered by the QIAquick Gel Extraction Kit (Qiagen) and further used for cycle sequencing with the PCR primers, applying the protocol of the BigDye Terminator v1.1 Cycle Sequencing Kit (Thermo Fisher Scientific, Dreieich, Germany). The DNA fragments were purified (Bioanalysis NucleoSEQ Kit, Macherey-Nagel), and 15 µL of the eluate was mixed with an equal volume of Hi-Di formamide (Thermo Fisher Scientific). The samples were sequenced either in one or both directions on a 3500 Genetic Analyzer device (Applied Biosystems/Hitachi, Darmstadt, Germany). Obtained sequences were edited with Geneious Prime software version 2021.0.1 (Biomatters, Auckland, New Zealand) and checked against NCBI GenBank (www.ncbi.nlm.nih.gov, last accessed on 11 December 2022). Edited sequences were deposited in GenBank and corresponding DNA samples were used for PCR validation.

### 2.4. Data Analysis and PCR Design

The real-time PCR was developed based on a multiplex in-house assay targeting the four taxa *C. obsoletus*, *C. scoticus*, *C. chiopterus*, and *C. dewulfi*. According to the reviewed consensus sequences from a recently published study [59], the PCR assay was updated and expanded to include the different clades of *C. obsoletus*. To do so, the specific consensus sequences of all subgenus *Avaritia* taxa were compared in a Geneious multiple alignment (Biomatters). Taking the resulting interspecific sequence differences into account, taxon-specific primers and probes were revised or newly designed according to common guidelines for PCR design (e.g., [84,85]). Since all genetic variants of the individual taxa had to be considered, degenerated bases were inserted at corresponding positions. Appropriate primer and probe sequences were checked in terms of melting temperature and hairpin structure or primer–dimer formation using the Oligo Analysis Tool (https://eurofinsgenomics.eu/en/ecom/tools/oligo-analysis/, last accessed on 11 December 2022) and analyzed with the NCBI BLAST Tool (https://blast.ncbi.nlm.nih.gov/Blast.cgi, 2.13.0 release, last accessed on 15 November 2022) for repetitive sequences. Selected primers (RP-Cartridge-Gold purified) and Taqman probes (HPLC purified) were ordered from Eurogentec (Kaneka Eurogentec S.A., Seraing, Belgium). The used CFX96 Touch Real-Time PCR Detection System (Bio-Rad, Feldkirchen, Germany) enables the simultaneous discrimination of a maximum of five targets in different fluorescence channels (FAM: 510–530 nm, HEX: 560–580 nm, TEX: 610–650 nm, Cy5: 675–690 nm, Quasar705: 705–730 nm), making it impossible to distinguish all six taxa in a single reaction well. Additionally, the PCR needed to be cost-effective and provide greater flexibility for the end-user. To address these requirements, the real-time PCR was designed in a modular manner, allowing the performance of each PCR in a singleplex format or their combination in various multiplex configurations, depending on the objectives of the scientific question.

### 2.5. Modular Real-Time PCR

Each master mix (singleplex or multiplex) was composed of 10 µL of 2× QuantiTect Multiplex PCR NoROX reagent (Qiagen), 0.375 µM of each primer, 0.125 µM of each probe, and 2 µL of DNA template, resulting in a total volume of 20 µL. The real-time PCR was performed in a 96-well format on CFX96 Touch Real-Time PCR Detection System (Bio-Rad) using the following thermoprofile: 15 min at 95 °C (activation of Taq polymerase), followed by 42 cycles of 30 s at 95 °C (denaturation), 30 s at 55 °C (primer annealing), and 30 s at 72 °C (primer elongation). The excited fluorescence of the probes was measured after each cycle at the end of the primer elongation step. The developed PCRs were tested in terms of their functionality, specificity, and capability for multiplexing and finally validated with genetically pre-identified (sequenced) material from field-collected culicoids or artificial COI genes [59,74].

## 3. Results

In this study, quantitative real-time PCRs targeting the mitochondrial (mt) COI gene were developed for the taxon-specific identification of six members belonging to the *Culicoides* subgenus *Avaritia*.

**Figure 1:**
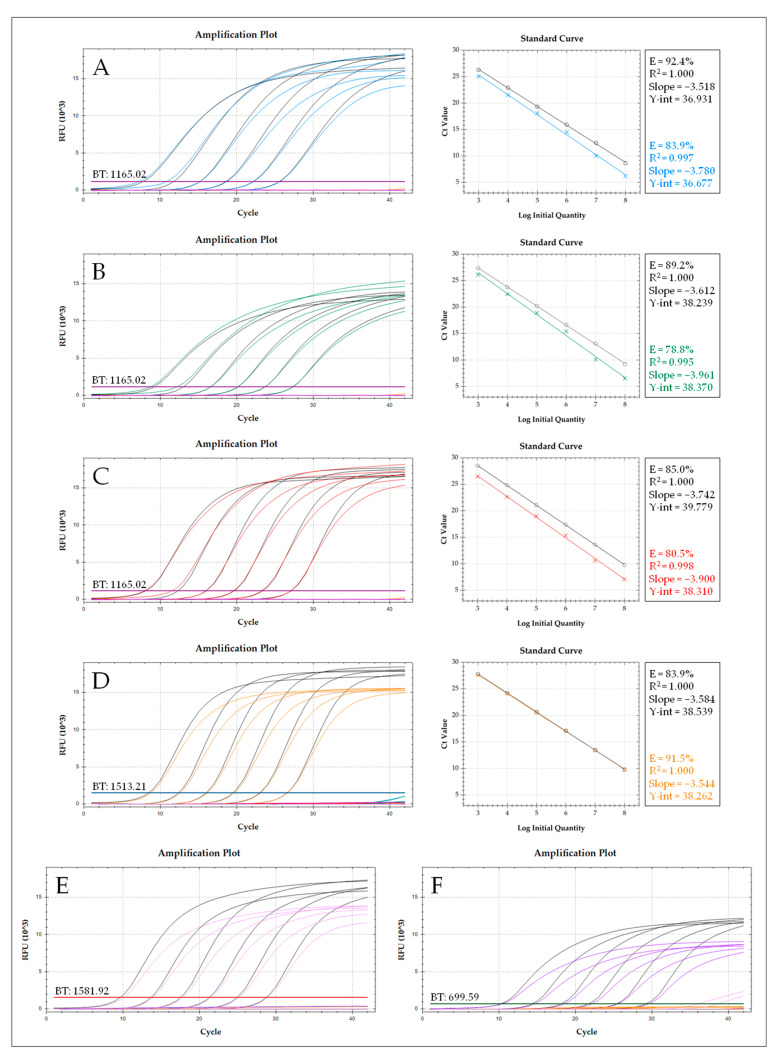
Direct comparison of the PCR performance between the sixplex PCR approach (colored) and the taxon-specific singleplex PCRs (black) through the simultaneous measurement of serial dilutions of the different target DNAs: *C. obsoletus* clade O1 ((**A**) blue, Atto647N), *C. obsoletus* clade O2 ((**B**) green, Atto647N), *C. obsoletus* clade O3 ((**C**) red, Atto647N), *C. scoticus* clade 1 ((**D**) orange, FAM), *C. chiopterus* ((**E**) pink, TEX), and *C. dewulfi* ((**F**) purple, HEX). BT: Baseline threshold in the corresponding detection channel. Calculated standard curves are given for *C. obsoletus* clade O1, *C. obsoletus* clade O2, *C. obsoletus* clade O3, and *C. scoticus* clade 1.

Taking the interspecific polymorphisms and intraspecific conserved regions of taxon-specific consensus sequences into account, 97 primers and 29 TaqMan probes were designed (Table S2). All primers and probes were pre-tested in terms of their functionality, PCR performance, and specificity to find the best working combination for the accurate detection of the different *Avaritia* taxa. Due to weak fluorescence signals, all Cy5 probes have been replaced by Atto647N labeled probes, which can be measured in the same detection channel (675–690 nm). The best-working primers and probes are summarized in Table 1. For four taxa, *C. obsoletus* clade O1, *C. obsoletus* clade O2, *C. obsoletus* clade O3, and *C. scoticus* clade 1, plasmids containing cloned COI target sequences [59] were used to generate standard curves and calculate the number of gene copies per single biting midge (Table S1). Of 142 individual DNA samples measured, only 57 were extracted following the same protocol (whole-body extraction with QIAamp DNA Mini Kit and an elution volume of 50 µL). Hence, these samples were used for the determination of the mt genome copy number per biting midge. The analysis revealed Ct values between 17.48 and 22.57 (mean Ct: 19.32), and a mean copy number of 1.2 × 10^6^ was calculated. Based on these findings, serial dilutions of cloned targets were prepared for each taxon (around 100- to 1000-fold lower and higher than the mean copy number per specimen) to evaluate the PCR performance of the different taxon-specific singleplex PCRs and compare the results to those obtained with the one-tube multiplex PCR approach (Figure 1). In the cases of *C. chiopterus* and *C. dewulfi*, no plasmids with cloned COI sequences were available at the time of assay design. Hence, serial dilutions of taxon-specific COI amplicons were prepared, which achieved similar Ct values to the plasmid standards of the other taxa.

Both the singleplex PCR and the multiplex PCR approach showed an almost similar performance. The calculated standard curves of all singleplex PCRs were linear over the selected measuring range with linear regression values (R^2^) of 1.000 and an amplification efficiency (E) between 83.9% and 92.4%. The R^2^s of the different taxa in the multiplex PCR (0.995–1.000) were comparable but the amplification efficiencies were, in most cases, lower (78.8–91.5%) than in the singleplex PCRs, which might be caused by undesired interactions of primers and probes or steric hindrance. The best results were achieved with the taxon-specific PCR of *C. scoticus* clade 1, which shows no difference in the overlay of calculated standard curves for both singleplex and multiplex PCR (Figure 1D). In general, no significant reduction in PCR sensitivity (∆Ct < 1) was determined between all singleplex PCRs and the multiplex approach (sixplex PCR), indicating no detrimental interactions of the taxon-specific primers in the PCR mixture. However, differences in the signal intensities (∆RFU) between +11.9% (*C. obsoletus* clade O2 PCR) and −22.8% (*C. dewulfi* PCR) were noticed for the multiplex PCR, and in some cases unspecific amplification signals were observed (Figure 1D,F), suggesting interactions (cross-reactivities) of respective TaqMan probes with other molecules of the PCR mixture or even the unspecific detection of non-target taxa.

**Figure 2:**
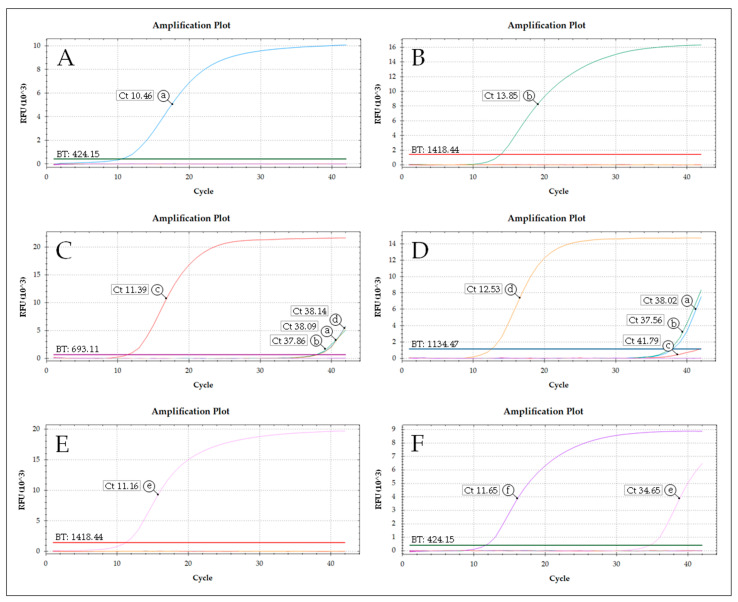
Determination of PCR cross-reactivity. Each singleplex PCR (**A**–**F**) was tested with 1 × 10^8^ copies of DNA of the various taxa (a–f) to simulate pool testing of 100 biting midges each: *C. obsoletus* clade O1 (PCR A, HEX, DNA a), *C. obsoletus* clade O2 (PCR B, TEX, DNA b), *C. obsoletus* clade O3 (PCR C, Atto647N, DNA c), *C. scoticus* clade 1 (PCR D, FAM, DNA d), *C. chiopterus* (PCR E, TEX, DNA e), and *C. dewulfi* (PCR F, HEX, DNA f). BT: Baseline threshold in the corresponding detection channel. RFU: Relative fluorescence units.

To confirm the observed non-specific amplification signals and determine the problem-causing molecules, each singleplex PCR was tested with 1 × 10^8^ mt genome copies (equivalent to the DNA amount of 100 specimens). Ct values between 10.46 and 13.85 were achieved for the specific DNAs and negative results were observed for the non-target taxa in the case of the PCR for *C. obsoletus* clade O1, *C. obsoletus* clade O2, and *C. chiopterus* (Figure 2A,B,E). However, unspecific DNA amplification (Ct values ≥ 34.65) was detected for the taxon-specific primers and probes of (i) *C. obsoletus* clade O3 with the DNAs of *C. obsoletus* clade O1, *C. obsoletus* clade O2, and *C. scoticus* clade 1 (Figure 2C); (ii) *C. scoticus* clade 1 with the DNAs of all three clades of *C. obsoletus* (Figure 2D); and (iii) *C. dewulfi* with the DNA of *C. chiopterus* (Figure 2F). Based on the Ct values obtained when testing a single biting midge (Ct < 20) and according to the observed cross-reactivities, a cut-off value was implemented, which considers samples with Ct > 30 as negative.

Taking this cut-off value into account, the diagnostic sensitivity (dSe) and specificity (dSp) of the multiplex PCR concept were determined for the various biting midge taxa through the analysis of genetically pre-identified specimens from field collections (Table S1). In total, 91 DNA samples of individual biting midges (whole specimens or body parts) were analyzed in a two-step PCR approach: First, the sixplex PCR was performed to identify specimens of *C. obsoletus* (no clade-separation), *C. scoticus* clade 1, *C. chiopterus*, and *C. dewulfi*. In a second step, the samples positive for *C. obsoletus* were further determined to the respective clades of *C. obsoletus* (clade O1, clade O2, or clade O3). Table 2 shows a summary of the obtained PCR results. Except for one DNA sample, which was positive for both *C. obsoletus* clade O2 (Ct 25.60) and *C. obsoletus* clade O1 (Ct 28.56), all target taxa of subgenus *Avaritia* biting midges were correctly identified, giving 100% dSe and 98.9% dSp.

The capability of the developed real-time PCR to analyze pools of unsorted biting midges was further assessed by examining the DNA of 36 *Culicoides* species and haplotypes representing seven additional subgenera endemic in the Palaearctic region (Table 3). The sixplex PCR was negative (Ct > 30) for 33 of the 36 taxa, giving a total dSp of 91.7%. However, two taxa of the subgenus *Avarita* (*C. montanus* and *C. scoticus* clade 2) were incorrectly detected as *C. obsoletus* (Ct 19.54) and *C. scoticus* clade 1 (Ct 19.97), respectively. In the case of unspecific detection of *C. montanus*, the sample was re-tested with the triplex PCR (*C. obsoletus* clade identification) for further verification of the cross-reactivity. The results were negative with the *C. obsoletus* clade O2 and *C. obsoletus* clade O3 PCRs, whereas a ‘false-positive’ result was observed with the *C. obsoletus* clade O1 PCR (Ct 22.91). A subsequent comparison of the taxon-specific consensus sequences revealed almost 100% identity in the primer and probe binding regions for *C. obsoletus* clade O1 and *C. montanus* (Table S3). Hence, samples identified as *C. obsoletus* clade O1 should be considered *C. obsoletus* clade O1/*C. montanus* and might be further analyzed by DNA barcoding. The same applies to *C. scoticus* clades 1 and 2. The analysis of the DNAs of both taxa displayed identical primer binding site sequences and only two base differences between the *C. scoticus* clade 1 probe (Csco-P-FAM) and the COI sequence of *C. scoticus* clade 2. Additionally, the synthetic DNA of *C. newsteadi* haplotype N2 (subgenus *Culicoides* Latreille) was incorrectly identified as *C. scoticus* clade 1 (Ct 27.12) if the sample was tested with the sixplex PCR or fourplex approach but was not detected with *C. scoticus* PCR alone. Further analysis of the false-positive PCR product was performed using gel electrophoresis followed by sequence comparison of all molecules applied in the fourplex PCR with the consensus sequence of *C. newsteadi* haplotype N2. It turned out that an 89 bp long PCR fragment was amplified with the forward primer of *C. obsoletus* clade O1 (CobsO1_F) and the reverse primer of *C. scoticus* (Csco_R) with the DNA of *C. newsteadi* haplotype N2, which was detected with the specific Taq-Man probe of *C. scoticus* clade 1 (Csco_P-FAM) (Table S3). Hence, if there is a high chance of finding specimens of *C. newsteadi* haplotype N2 in field collections, a morphological pre-identification of the samples to the group level (separation of specimens according to Obsoletus Group and Pulicaris Complex via wing pigmentation) or the usage of the *C. scoticus* clade 1 PCR in a multiplex approach without the *C. obsoletus* clade O1 PCR is recommended.

To confirm the applicability of the real-time PCR for the direct measurement of unsorted insect samples (*Culicoides* plus bycatch), genetically pre-identified DNA samples of 15 non-culicoid Diptera (Table 4) were analyzed with the developed multiplex PCR (sixplex). For three taxa, Ct values > 35.8 were obtained with the PCRs for *C. obsoletus* and *C. dewulfi*, despite a considerable number of mismatches between the primer and probe sequences and the non-culicoid DNA (Table S4). However, all tested samples were negative if the mentioned cut-off value was applied. Hence, if the observed cross-reactivities are considered, the developed PCR approach has great potential to be used for the specific detection of *Avaritia* biting midge taxa in unsorted UV-light catches.

## 4. Discussion

In the past two decades, PCR-based methodologies have increasingly been implemented in the discipline of entomology as standard diagnostic tools, especially when dealing with sibling species of, e.g., culicoid vectors [73,87]. Several PCR tests have been developed to distinguish isomorphic specimens of the widespread Obsoletus Group, the main vectors of BTV, SBV, and EHDV in central Europe. Most of these assays include time-consuming gel electrophoresis steps [57,69,76,77,88,89] and are inefficient at analyzing large quantities of samples that usually accumulate during entomological surveillance and monitoring activities. Given these deficiencies, the knowledge about the geographic distribution and ecology of vector species is still fragmentary.

In this study, a high-throughput multiplex real-time PCR was successfully developed that enables the quantitative detection of vector species within the *Culicoides* subgenus *Avaritia*, including the clades of *C. obsoletus*. A major challenge was the acquisition of sufficient DNA material of the various *Culicoides* taxa. Therefore taxon-specific PCR development and its verification were restricted, and synthetic DNA had to be used in some cases. Additionally, the PCR was meant to consider the sequences of all genetic variants (haplotypes) of the target taxa across a wide geographical area to ensure universal applicability. To obtain the necessary genetic data, publicly accessible databases such as GenBank are available. However, sequences deposited in databases should be used with caution due to a considerable number of incorrectly annotated entries. Recently, Dähn et al. [59] successfully developed a conventional PCR for the different taxa of the subgenus *Avaritia* based on reviewed and scrutinized COI sequences from GenBank. This sequence dataset was successfully used in this study to design taxon-specific primers and probes.

The developed real-time PCR achieved a diagnostic sensitivity (dSE) of 100% due to the high-copy properties of the COI gene. Notwithstanding, the exact quantification of the target DNA turned out to be difficult due to inhomogeneous DNA extraction. The biting midge size proved to be particularly disadvantageous as during extraction of an individual midge, some parts were not completely lysed. This likely explains the obtained variations in the Ct-values when analyzing single midges (19.32 ± 4 SD).

High sensitivity also indicates highly efficient PCR amplification, which can even amplify the smallest traces of DNA (a few nanograms) [90]. Millions to billions of copies are rapidly produced [91], particularly when generating short amplicons, as is common in real-time PCRs (50–150 bp) [92]. This can readily lead to carryover contamination from previous PCRs [93]. When assessing the specificity of the developed PCR, this contamination was particularly problematic. Despite preventive measures, such as spatial separation of the master mix preparation and DNA sample addition, amplification signals were observed for several non-target taxa. For example, the DNA of *C. newsteadi* haplotype N1 (Ct 36.09) was detected with the taxon-specific PCR of *C. dewulfi*, even though the probe sequence (26 bp in length) exhibited 11 mismatches compared to the sequence of this DNA sample (c.f. Table S3). Since numerous PCR developments and sequencing efforts of various taxa of the subgenus *Culicoides* [74] and *Avaritia* [59] were conducted simultaneously in the same laboratory facilities, contamination appears to be the most likely explanation. This assumption could be confirmed by repetitive measurement of the sample, which only exceptionally resulted in positive detection of the non-target taxon. A cut-off value was successfully established to differentiate between non-specific and specific amplification signals in the developed PCR assay.

Other false-positive results that occurred are associated with issues arising from unclear phylogeny of certain *Culicoides* taxa. For instance, the taxon-specific PCR of *C. obsoletus* clade O1 developed in this study also detected *C. montanus.* Several authors suggested that *C. montanus* represents a genetic variant of *C. obsoletus* clade O1 [58,59,60], so its status as a distinct species remains controversial. Due to the extremely high degree of sequence homology between *C. obsoletus* and *C. montanus*, it has not yet been possible to distinguish both species when using the COI gene as a molecular marker for PCR development [59,62,77,88,94]. Interestingly, despite the even higher sequence similarity in ribosomal ITS1 (98.3%) and ITS2 (98.6%) spacers, as compared to the mt COI gene (97.2%), some authors have been able to discriminate *C. montanus* from *C. obsoletus* by those genetic markers [57,78]. Additionally, *C. obsoletus* and *C. montanus* specimens can easily be separated by experienced entomologists based on the morphology of both the palp and the long trichodea [82], without any overlapping between both species. The same genetic and morphological discrepancy is observed for *C. scoticus* clade 1 and *C. scoticus* clade 2 [59,82]. Assuming that *C. montanus* and *C. scoticus* clade 2 are not distinct species, the calculated diagnostic specificity of the multiplex PCR (sixplex) would need to be adjusted to more than 97.2%. However, it should be noted that, due to the limited availability of sample material, only the DNA of a single specimen or synthetic DNA representing the respective taxa has been tested so far. Further comparative morphological and genetic analyses of individual specimens of respective taxa are needed to clarify the status of *C. montanus* and *C. scoticus* clade 2 and correctly interpret the obtained false-positive results of the developed real-time PCR.

The only verified cross-reactivity below the defined cut-off value was observed with the taxon-specific PCR for *C. scoticus* clade 1 when tested against the DNA of *C. newsteadi* haplotype N2. Although the individual PCRs were specifically designed, limitations in the specificity of individual molecules can accumulate in the multiplex PCR, resulting in the generation and detection of non-specific amplicons. In this study, the forward primer of *C. obsoletus* clade O1 and the reverse primer of *C. scoticus* clade 1 amplified a PCR fragment with the DNA of *C. newsteadi* haplotype N2, which was detected with the probe of *C. scoticus* clade 1. Hence, the *C. scoticus* clade 1 PCR should not be used in combination with the taxon-specific PCR for *C. obsoletus* clade O1 if unsorted *Culicoides* are analyzed. This issue highlights the challenges of multiplex development, especially when the targeted DNA region offers limited space and possibilities for positioning several PCRs. The design of sequencing primers capable of amplifying a longer genomic marker and providing greater flexibility for the positioning of primers and probes would be beneficial for future PCR developments. Alternatively, minor groove binding probes could be used in A/T-rich gene regions to enhance PCR specificity [62]. The side-directed insertion of mismatch bases into primers or probes, designed to not interfere with the detection of the target taxon [59,74], was also tested in this study. However, its application in quantitative real-time PCR proved to be challenging due to observed shifts in Ct values and reduced probe signal intensities, which affected the accuracy of genome copy number calculations.

Despite the challenges mentioned, the developed real-time PCR has demonstrated high reliability when testing DNA extracts of single specimens or body parts (thorax, abdomen). The latter is a major advantage over less-sensitive methods such as DNA barcoding, which often require entire midges for the analysis, making morphological re-analysis impossible. The developed multiplex PCR demonstrated successful quantification of its target taxa with a PCR performance comparable to the singleplex PCR approach. The definition of an appropriate cut-off value even allowed the correct determination of the selected target taxa in mixed pools of up to 100 culicoid specimens or bycatch, which is a prerequisite for a high-throughput screening approach needed for surveillance studies. Thus, the developed PCR provides significant potential for monitoring the European vector species and facilitates the implementation of preventive measures to control culicoid-borne diseases such as BTD, Schmallenberg disease, and epidemic hemorrhagic disease of cervids.

## 5. Conclusions

Culicoid biting midges commonly achieve high population densities, resulting in tremendous numbers of specimens being captured in UV-light traps. This presents a significant challenge in entomological surveillance studies. Isomorphic species of the Obsoletus Group often comprise a substantial proportion of the collected specimens. The real-time PCR developed in this study is the first of its kind capable of efficiently processing such sample sizes while quantifying the relative proportions of all known taxa within the Obsoletus Group in Europe. Although further validation is needed to evaluate the method’s robustness, especially for analyzing pools of unsorted insect catches, the PCR has demonstrated superior efficiency and reliability compared to conventional gel-based PCRs. This advancement marks a significant step toward the routine collection of data on the distribution of ceratopogonid vector species, enabling improved assessment of current and future risk scenarios. Such capabilities are increasingly critical considering the growing globalization and climate change, allowing the timely implementation of countermeasures.

## Figures and Tables

**Table 1:** List of specific primers and probes designed for six taxa of the subgenus *Avaritia*. The PCR approach is designed in a modular manner: All PCRs can be either used alone (singleplex) or combined in different single-tube multiplex approaches (triplex, fourplex, sixplex). The sixplex PCR allows the differentiation of *C. obsoletus* (all three clades labeled with Atto647N probe), *C. scoticus* clade 1 (FAM), *C. chiopterus* (TEX), and *C. dewulfi* (HEX). For further identification of *C. obsoletus* positive samples to the clade level, a second PCR must be performed through specific signals of the following fluorophores: *C. obsoletus* clade O1 (HEX), *C. obsoletus* clade O2 (TEX), and *C. obsoletus* clade O3 (Atto647N). If there is no need to determine *C. chiopterus* and *C. dewulfi*, the samples can be directly analyzed in a one-step approach either for *C. obsoletus* clades O1 to O3 (triplex) alone or in combination with *C. scoticus* (fourplex).

Taxon	Oligo Name	5′-Modification	Sequence (5′ > 3′)	3′-Modification	Amplicon Size (bp)	Sixplex	Fourplex	Triplex	**Singleplex**
*C. obsoletus* clade O1	CobsO1_F	none	AGAAAAYGGRGCAGGAACC	none	89	x	x	x	x
	CobsO1_R	none	AAATAGCCAAATCTACAGAAG	none		x	x	x	x
	CobsO1_P-Atto	Atto647N	CCCTCCCTTTCRTCTAATATCTCT	BHQ-2		x	-	-	x
	CobsO1_P-HEX	HEX	CCCTCCCTTTCRTCTAATATCTCT	BHQ-1		-	x	x	x
*C. obsoletus* clade O2	CobsO2_F	none	AGCCGTTAATTTTATTACAACC	none	127	x	x	x	x
	CobsO2_R	none	TAATACAGGTAAAGATAGYAGG	none		x	x	x	x
	CobsO2_P-Atto	Atto647N	ACGATCATATGGAATAAMTTTCGATC	BHQ-2		x	-	-	x
	CobsO2_P-TEX	TEX	ACGATCATATGGAATAAMTTTCGATC	BHQ-2		-	x	x	x
*C. obsoletus* clade O3	CobsO3_F	none	GCTCTATTTTAGGTGCTGTT	none	107	x	x	x	x
	CobsO3_R	none	AGTAATTAATACAGATCATACG	none		x	x	x	x
	CobsO3_P-Atto	Atto647N	TATTATCAATATRCGATCATACGGGA	BHQ-2		x	x	x	x
*C. scoticus* clade 1	Csco_F	none	CACTTTATTATTAATTAGAAGTTTAGTT	none	116	x	x	-	x
	Csco_R	none	AAATTGCTAAGTCAACTGAGG	none		x	x	-	x
	Csco_P-FAM	FAM	ACCCTCCACTTTCAGCAAATGTCT	BHQ-1		x	x	-	x
*C. chiopterus*	Cchi_F	none	CCCTGATATAGCTTTTCCA	none	86	x	-	-	x
	Cchi_R	none	CTAAGCTACTTAYTAATAGTAG	none		x	-	-	x
	Cchi_P-TEX	TEX	TGAATACTRCCRCCCTCTATCACC	BHQ-2		x	-	-	x
*C. dewulfi*	Cdew_F	none	ACAATCATTAATATACGACCAA	none	118	x	-	-	x
	Cdew_R	none	TARCTCCTGCTAAAACTGGA	none		x	-	-	x
	Cdew_P-HEX	HEX	CACAGCTATTCTTTTACTTCTGTCAC	BHQ-1		x	-	-	x

x: Applicable. -: Not applicable.

**Table 2:** Determination of the diagnostic sensitivity (dSe) and specificity (dSp) of the developed real-time PCR for the various biting midge taxa. A total of 91 genetically pre-identified (sequenced) DNA samples of single specimens were initially tested with the sixplex PCR. Samples detected with Atto647N-labeled probe (*C. obsoletus*) were further analyzed with a triplex PCR to differentiate the three *C. obsoletus* clades.

Taxon	Samples Tested (n) ^1^	Sixplex PCR (n) ^2^	Triplex PCR (n) ^2^	dSe	dSp
*C. obsoletus* clade O1	39	70	39	39/39 (100%)	38/39 (97.4%)
*C. obsoletus* clade O2	28		29 ^3^	28/28 (100%)	28/28 (100%)
*C. obsoletus* clade O3	3		3	3/3 (100%)	3/3 (100%)
*C. scoticus* clade 1	10	10	-	10/10 (100%)	10/10 (100%)
*C. chiopterus*	4	4	-	4/4 (100%)	4/4 (100%)
*C. dewulfi*	7	7	-	7/7 (100%)	7/7 (100%)
Total	91	91	71	91/91 (100%)	91/92 (98.9%)

^1^ Taxon-specific DNA samples of single specimens, pre-identified with COI barcoding. ^2^ Number of samples with a positive result. Cut-off value: Ct < 30 = positive. ^3^ OQ941557 was positive for *C. obsoletus* clade O2 (Ct 25.60) and *C. obsoletus* clade O1 (Ct 28.56). -: Not applicable.

**Table 3:** Cross-reactivity of the newly developed real-time PCR against other *Culicoides* taxa (n = 36). The DNAs of single specimens or 1 × 10^6^ copies of the artificial COI gene of each taxon were tested with the sixplex PCR approach and, if necessary, the triplex PCR to determine the specific clade of *C. obsoletus*. The lowest Ct values are indicated in brackets and were specified by taxon-specific PCRs. Ct values > 30 (above the cut-off) were considered negative.

Subgenus	Taxon	GenBank Accession No.	Real-Time PCR Result
*Avaritia* Fox, 1955	*C. imicola* ^2^	OQ789072	negative (Ct 36.79) ^4^
	*C. montanus* ^2^	OQ789074	positive (Ct 19.54) ^5^
	*C. sanguisuga* ^2^	MK760238	negative
	*C. scoticus* clade 2 ^2^	OQ789084	positive (Ct 19.97) ^6^
	*C. sinanoensis* ^2^	MK760244	negative
*Beltranmyia* Vargas, 1953	*C. salinarius* ^2^	OQ789083	negative
*Culicoides* Latreille, 1809	*C. boyi* ^3^	-	negative
	*C. bysta* ^3^	-	negative
	*C. cryptipulicaris* ^3^	-	negative
	*C. delta* ^2^	OQ789035	negative
	*C. fagineus* haplotype F1 ^3^	-	negative
	*C. fagineus* haplotype F2 ^2^	OQ789036	negative
	*C. flavipulicaris* ^3^	-	negative
	*C. grisescens* haplotype G1 ^2^	OQ789037	negative (Ct 38.25) ^7^
	*C. grisescens* haplotype G2 ^2^	OQ789038	negative (Ct 36.27) ^7^
	*C. kalix* ^3^	-	negative
	*C. lupicaris* haplotype L1 ^2^	OQ789039	negative
	*C. lupicaris* haplotype L2 ^2^	OQ789041	negative
	*C. newsteadi* s.s. ^3^	-	negative (Ct 32.40) ^6^
	*C. newsteadi* haplotype N1 ^2^	OQ789045	negative (Ct 36.09) ^4^
	*C. newsteadi* haplotype N2 ^3^	-	positive (Ct 27.12) ^6^
	*C. newsteadi* haplotype N3 ^2^	OQ789048	negative
	*C. pulicaris* ^2^	OQ789058	negative
	*C. punctatus* ^2^	OQ789064	negative
	*C. selandicus* ^2^	OQ789052	negative
	*C. subfagineus* ^3^	-	negative
*Monoculicoides* Khalaf, 1954	*C. riethi* ^2^	OQ789081	negative
*Sensiculicoides* Shevchenko, 1977	*C. alazanicus* ^2^	OQ789067	negative
	*C. festivipennis* ^2^	OQ789070	negative (Ct 34.85) ^7^
	*C. griseidorsum* ^2^	OQ789071	negative
	*C. kibunensis* ^2^	OQ789073	negative
	*C. pictipennis* ^2^	OQ789079	negative (Ct 34.72) ^7^
	*C. poperinghensis* ^2^	OQ789080	negative
*Silvaticulicoides* Glukhova, 1977	*C. achrayi* ^2^	OQ789066	negative
*Wirthomyia* Vargas, 1973 ^1^	*C. riouxi* ^2^	OQ789082	negative
Unplaced	*C. pallidicornis* ^2^	OQ789078	negative (Ct 34.63) ^4^
dSp (other *Culicoides* taxa):	n = 36	33/36 (91.7%)	

^1^ According to the latest world catalog of biting midges [86], this species is unplaced, but—based on male and female morphology—should be assigned to the subgenus *Wirthomyia*. ^2^ DNA extracts of single specimens. ^3^ 1 × 10^6^ copies of the artificial COI gene [74]. ^4^ *C. dewulfi* (HEX) in sixplex PCR. ^5^ *C. obsoletus* clade O1 (HEX) in triplex PCR. ^6^ *C. scoticus* clade 1 (FAM) in sixplex PCR. ^7^ *C. obsoletus* (Atto647N) in sixplex PCR. -: Not applicable.

**Table 4:** Cross-reactivity of the newly developed real-time PCR against non-culicoid ‘dipteran species’ (n = 15). The DNA extract of one specimen each, representing either a species or a genus, was tested. All samples were analyzed with the sixplex PCR approach. Ct values are indicated in brackets and were specified by taxon-specific PCRs. Ct values > 30 (above the cut-off) were considered negative.

Taxon/Genus	GenBank Accession No.	Real-Time PCR Result
*Alluaudomyia* spec.	PP110213	negative
*Camptocladius stercorarius*	PP110214	negative
*Chironomus lugubris*	PP110215	negative
*Clogmia albipunctata*	PP110216	negative (Ct 36.40) ^1^
*Desmometopa sordida*	PP110227	negative
*Forcipomyia* spec.	PP110217	negative (Ct 35.83) ^1^
*Nemotelus notatus*	PP110218	negative
*Nilotanypus dubius*	PP110219	negative
*Physiphora alceae*	PP110220	negative
*Psychoda cinerea*	PP110221	negative
*Sepsis violacea*	PP110222	negative
*Smittia* spec.	PP110223	negative
*Spelobia luteilabris*	PP110224	negative
*Sphaerocera curvipes*	PP110225	negative
*Tephrochlamys rufiventris*	PP110226	negative (Ct 36.89) ^2^
dSp (other Diptera taxa):	n = 15	15/15 (100%)

^1^ *C. dewulfi* (HEX). ^2^ *C. obsoletus* (Atto647N).

## Data Availability

Publicly available datasets used in this study are listed in the Supplementary Material of reference [57]. The respective data can be found in GenBank at: https://www.ncbi.nlm.nih.gov/genbank/ (accessed on 15 November 2022). Data additionally created in this study are deposited in the same data repository with the following accession numbers: *Desmometopa sordida* (PP110227).

## Supplementary Materials

The following supporting information can be downloaded at https://www.mdpi.com/article/10.3390/insects16050500/s1, Table S1: List of DNA extracts used to determine the diagnostic sensitivity (dSe) and specificity (dSp) of the developed real-time PCRs for their different target taxa; Table S2: List of all primers and probes tested for the development of the taxon-specific PCRs; Table S3: Cross-reactivity of the newly developed real-time PCRs against other *Culicoides* taxa; Table S4: Cross-reactivity of the newly developed real-time PCRs against selected non-culicoid insect taxa.

## Abbreviations

The following abbreviations are used in this manuscript:

28S rDNA	structural ribosomal RNA (large subunit)
bp	base pairs
BT	baseline threshold
BTD	bluetongue disease
BTV	bluetongue virus
CAD	carbamoyl-phosphate synthetase 2
COI	mitochondrial cytochrome c oxidase subunit I
Ct	cycle threshold
Cy5	cyanine5
cytb	mitochondrial cytochrome b
DNA	deoxyribonucleic acid
dSe	diagnostic sensitivity
dSp	diagnostic specificity
EHD	epizootic hemorrhagic disease
EHDV	epizootic hemorrhagic disease virus
FAM	6-carboxyfluorescein
HEX	hexachlorofluorescein
HPLC	High-performance liquid chromatography
ITS-1	internal transcribed spacer 1
ITS-2	internal transcribed spacer 2
mt	mitochondrial
NCBI	National Center for Biotechnology Information
PCR	polymerase chain reaction
RFU	relative fluorescence unit
s.s.	sensu stricto
SBD	Schmallenberg disease
SBV	Schmallenberg virus
TEX	Texas Red (sulforhodamine 101 acid chloride)
